# Profiles of Family Functioning and Their Associations with Physical and Psychological Health Outcomes

**DOI:** 10.3390/famsci2020014

**Published:** 2026-05-12

**Authors:** Jonathan G. Kimmes, Thomas Ledermann, Shannon Montgomery, Nicholas Triplett, Joseph G. Grzywacz

**Affiliations:** 1 Department of Human Development and Family Science, Florida State University, Tallahassee, FL 32304, USA; 2 College of Health and Human Sciences, San José State University, San José, CA 95192, USA

**Keywords:** couples, family functioning, physical health, psychological health, latent profile analysis

## Abstract

Using a pattern-centered approach, this investigation sought to identify latent subgroups of family functioning and evaluate their associations with health outcomes. A latent profile analysis was conducted using data from the National Study of Midlife Development in the United States (MIDUS). The sample comprised 5923 married adults between 25 and 74 years old who responded to items measuring family support, family strain, spousal support, spousal strain, and spousal agreement. These aspects of family functioning were used to identify four latent classes: Low Functioning (5.0%), Moderate Support with Elevated Strain (31.7%), High Support with Elevated Strain (13.7%), and High Functioning (49.6%). Compared to the other classes, individuals in the High Functioning class had greater psychological well-being and were less likely to have a chronic physical condition. The findings of this study highlight the importance of the systemic context of families in understanding the role of close relationships in health.

## 1. Profiles of Family Functioning

Family relationships are undoubtedly important for physical and psychological health (Berge et al., 2006; Pietromonaco & Collins, 2017; Smith, 2019), regardless of whether the relationships are among intimate partners or among same- or intergenerational others (e.g., siblings, parents, children). Like all social relationships, family relationships can be sources of support (e.g., emotional, instrumental, financial) that provide clear benefits for health and well-being (Berge et al., 2006). However, family relationships can also be a source of burden and strain (Burman & Margolin, 1992; Walen & Lachman, 2000), such as when a child’s or partner’s illness requires caregiving or when a parent’s expectations for spending the holidays together misaligns with personal goals.

Family functioning comprises support and strain, and some researchers have suggested that disagreement, or level of disharmony, is another key aspect of family functioning (e.g., Keown & Woodward, 2002). Although family functioning is a multidimensional construct, there is no clear consensus about what specific variables comprise the dimensions of family functioning. Regardless of how different researchers have defined the dimensions of family functioning, they are rarely investigated in unison using a person-centered approach. That is, researchers frequently study the “independent effects” of support and strain. However, when considered conceptually and theoretically, the “independent effects” variable-based approach demands questioning. By adopting a person-centered approach, it may be possible to identify specific subgroups that share similar levels of support and strain, as well as explore how those subgroups relate to specific physical and psychological health outcomes, which may ultimately inform the development of intervention programs that focus on strengthening family relationships. The present study takes a closer look at latent subgroups and aims to advance the understanding of different patterns of family functioning in married individuals from a national representative study.

One of the theoretical underpinnings for the present study is general systems theory, which emphasizes that independent observation of all of the “parts” of a system is not tantamount to observation of the whole system and which can be used to recognize and account for the interdependencies within family relationships and family subsystems (Bateson, 1972; von Bertalanffy, 1968; White & Klein, 2002). Experiences of support and strain in one family relationship, like the marital union, are likely separate but perhaps related to other experiences of support and strain from another family relationship (e.g., parents or siblings). In this way, support and strain across specific family relationships likely create a complex system of intertwining relations in the family as a whole. Therefore, in order to better understand the role of family relationships in the context of adult health, it is necessary to explore patterns of configurations of support and strain within family relationships.

The interdependence and synchrony of support and strain inherent in family relationships may be best characterized with a pattern-centered approach, as opposed to a variable-centered strategy, to identify different subgroups of family functioning. Results from previous studies provide support for the use of person-centered approaches in identifying subgroups of family functioning. In one study, a latent class analysis was used to identify three classes of functioning in 234 families with a child in kindergarten: cohesive, enmeshed, and disengaged (Sturge-Apple et al., 2010). In another study, a latent profile analysis was conducted on family functioning in a sample of 273 military families, and a four-group solution was selected as the optimal one (i.e., balanced, unbalanced, mid-range, and rigidly balanced; Oshri et al., 2015). In these studies, profiles of family functioning were linked with key outcomes, such as internalizing symptoms, mental health, and physical health. However, to our knowledge, subgroups of family functioning have not been identified using data from a nationally representative sample. Building on this emerging literature, we endeavored to delineate subgroups of family functioning among married adults using nationally representative data and then explore variation in physical and mental health outcomes across subgroups of family functioning.

The life course perspective also informs the present study. Specific time periods and their attendant sociocultural and economic realities can play a role in the dynamics of family relationships, which, in turn, affect the health of those within the family system (Hagestad, 2002). Cross-sectional analysis of a single time point can provide insight into the association between marriage, family life, and health, but it only provides a snapshot into how certain factors may affect health at one period in history. Investigation of individuals from different historical contexts can help identify continuity and change in the pattern of interdependency and synergy in experiences of support and strain across distinct family relationships. Furthermore, the use of individuals from two different historical contexts also allows the study of possible variation in how family functioning relates to family members’ well-being (i.e., “cohort effects”).

## 2. Dimensions of Family Functioning

Family functioning is a multidimensional concept. Support and strain in family relationships, for example, are crucial and distinct aspects of family functioning (Walen & Lachman, 2000). Although they are related constructs, the lack of one does not suggest the other is abundant, and individuals may simultaneously feel strain and support from a specific family member. Furthermore, support and strain have been linked to a range of outcomes, but studies have produced conflicting results. For example, one study demonstrated that partner strain but not partner support was associated with increased health problems (Walen & Lachman, 2000). Another study found a significant positive association between spousal support and self-rated health, as well as a significant inverse association between spousal support and functional limitations (Ryan et al., 2014); interestingly, the researchers did not find a significant association between spousal strain and health outcomes. Further research, therefore, is needed to help elucidate the links between strain, support, and health outcomes.

Research involving family functioning often fails to account for the contribution of the marital relationship, and, conversely, research that focuses on marital functioning frequently ignores the importance of broader family relationships (K. Birditt & Antonucci, 2008). Like broader family relationships, the marital relationship is a source of support and strain. Although the support and strain experienced in the marriage may not resemble the functioning of broader family relationships, marital functioning is linked to and a part of the functioning of the family overall. Examining functioning within the marriage separately from the functioning of the family, therefore, does not account for how marital functioning and family functioning operate together. For example, having multiple stressful family relationships may motivate individuals to cultivate a better marriage that can serve as an oasis in the midst of troubled family relationships, ultimately leading to higher levels of support experienced in the marital relationship. Although there have been recent calls for more research involving the differential effects of specific types of family relationships, this imperative does not account for the systemic, interdependent nature of the family context. The use of person-centered approaches, including latent profile analysis, is consistent with the notion that the marital relationship and other family relationships are interdependent. As Zyphur (2009) stated, “any areas of research normally prompting a regression mindset but involving the study of variables that could be considered as a coherent system may be recast in a theoretically useful way by adopting an LPA mindset” (p. 683).

## 3. Dimensions of Family Functioning and Psychological and Physical Health

Family functioning has been repeatedly shown to have a positive association with quality of life and markers of mental health (e.g., Ali & Malik, 2015; Cheng et al., 2017). Higher family functioning has also been linked to better overall physical health, as well as indicators of physical well-being (e.g., Cepukiene & Celiauskaite, 2020; García-Huidobro et al., 2012; Oshri et al., 2015). It is important to note that intimate partnerships and broader family relationships may differ in terms of the role they play in individual well-being, as they have been shown to have unique links to health outcomes (Woods et al., 2020).

Spousal support and strain are two specific components of family functioning that have also been evaluated in terms of their relationship with psychological and physical health outcomes. Spousal support and strain may have different associations with health outcomes. In fact, one study demonstrated that partner support and strain are differentially linked to physical and mental health (Barr & Simons, 2014). Specifically, strain but not support was significantly associated with both physical and mental health. Other research has found that spousal support and strain predicted psychological but not physical health (Hung et al., 2019), and that spousal support but not spousal strain was linked to physical health (Ryan et al., 2014). Although the dimensions of family functioning and overall family functioning are associated with health outcomes, utilizing a pattern-based approach to research family functioning may help advance understanding of how the interconnectedness of family relationships can promote or compromise health.

## 4. Time Period Processes in Relation to Family Functioning and Health

Life course epidemiology aims to build and test theoretical models of health outcomes that acknowledge interconnections between historical and personal time. Ideas from life course epidemiology can benefit family science by considering how outcomes of family life (e.g., individual health and well-being) vary within a changing world (Mishra et al., 2015). Furthermore, there is a need to explore how family dynamics are similar and different based on the time period. Particular patterns of family functioning may be apparent at one time period, but these patterns may not be found at another time period. Alternatively, the patterns of family functioning may not change between time periods, but the proportion of families that exhibit specific patterns of family functioning may change. Historical changes in the structure of families, as well as cultural and economic shifts, may change the way in which the dimensions of family functioning tend to constellate within families. In order to better understand patterns of family functioning, it is important to explore potential historical changes in these patterns because families and their interactions may change based on social, cultural, or economic shifts they experience.

## 5. Present Study

The purpose of this study was to advance understanding of how aspects of family functioning play a role in variation in physical and psychological health. To achieve this goal, we used data from the National Study of Midlife Development in the United States (MIDUS). Data from two cohorts were used; the first cohort provided data between 1995 and 1996, and the second provided data between 2011 and 2014. These repeated cross-sectional data were used in the present study to accomplish three primary aims. The first aim was to identify latent subgroups of individuals that share similar configurations of the following aspects of family functioning: spousal support, spousal strain, spousal disagreement, generalized family support, and generalized family strain. In the second aim, we delineated variation in psychological and physical health outcomes by the latent subgroups of family functioning. The third aim was to compare two cohorts based on the proportion of each sample that belonged to each of the subgroups of family functioning.

Because the present study will involve a sample of middle-aged adults, the findings will contribute to the understanding of a pivotal but understudied period in the life course. During midlife, relationships and other life experiences are consequential for outcomes in later life (Lachman et al., 2015), but more research that directly investigates midlife is necessary to improve “our understanding of this largely understudied age period and inform interventions to promote their well-being” (Lachman, 2015, p. 333).

## 6. Method

### 6.1. Sample and Procedure

Data from the two cohorts were drawn from the first wave of the Midlife in the United States (MIDUS 1) study and from the MIDUS Refresher (MIDUS-R). The MIDUS 1 study comprised over 7000 English-speaking adults in the United States between 25 and 74 years old, and the first wave of data for the first cohort was collected in 1995 and 1996. This cohort will be referred to as “cohort 1”. The second cohort used data from the MIDUS-R, which was made up of approximately 3600 individuals recruited between 2011 and 2014 (C. Ryff et al., 2016). This cohort will be referred to as “cohort 2”. The two cohorts are demographically identical.

In the present study, the two samples were limited to individuals from cohort 1 (*n* = 4244) and from cohort 2 (*n* = 1679) who reported being currently married. Of those from cohort 1, 51.3% were male and 48.7% were female, and of the individuals from cohort 2, 54.0% were male and 46% were female. Among individuals in cohort 1 and 2, the average age was 47.5 years (*SD* = 13.4) and 47.5 years (*SD* = 13.6), respectively. Most participants reported their race as White (80.3% and 80.9%, respectively). In terms of education, 90.0% of cohort 1 and 90.4% of cohort 2 had at least graduated from high school, and about two-thirds of the participants from each cohort had at least some college experience. Institutional review board approval was not required for this study because we used de-identified, publicly available, secondary data.

### 6.2. Measures

Spousal support and strain and generalized family support and strain. Spousal support, spousal strain, family support, and family strain were measured using six items each (Walen & Lachman, 2000), and each item was rated on a 4-point scale. Example items from the measures of support and strain include: “How much does your spouse or partner appreciate you?” (spousal support); “How often does he or she criticize you?” (spousal strain); “Not including your spouse or partner, how much do members of your family really care about you?” (family support); “How often do they get on your nerves?” (family strain). For each of the four variables, the six items were averaged. For the variables measuring support, items were recoded so that higher scores reflect higher support. For the variables measuring strain, items were recoded such that higher scores indicate lower strain. Coefficient *α* was 0.82 for family support, 0.80 for family strain, 0.86 for spouse support, and 0.81 for spouse strain.

Spousal disagreement. Spousal disagreement was measured using three items on a four-point scale ranging from 1 = a lot to 4 = not at all (Walen & Lachman, 2000). Participants were asked to indicate how much they disagree with their spouse on issues involving: money, household tasks, and leisure activities. All items were reverse-coded so that higher scores indicate greater disagreement. Coefficient *α* was 0.68.

Psychological well-being. Six dimensions of psychological well-being (i.e., self-acceptance, autonomy, personal growth, positive relations with others, purpose in life, environmental mastery) were measured using three items each (C. D. Ryff & Keyes, 1995). Each item was on a seven-point scale (1 = strongly agree to 7 = strongly disagree), and a score for each of the six subscales was constructed by calculating the sum of each set of items. Example items from the six subscales include: “I like most parts of my personality” (self-acceptance), “I tend to be influenced by people with strong opinions” (autonomy), “For me, life has been a continuous process of learning, changing, and growth” (personal growth), “Maintaining close relationships has been difficult and frustrating for me” (positive relations with others), “I sometimes feel as if I’ve done all there is to do in life” (purpose in life), “I am good at managing the responsibilities of daily life” (environmental mastery). Items were coded such that higher scores indicate higher levels of well-being. Coefficient alphas for the six subscales range from 0.36 to 0.59. The low alpha levels for the six dimensions are worth noting; however, when a measure has sufficiently covered the content of the domain of interest, comprises only a few items, and has reasonable unidimensionality, it has been argued that relatively low alphas may be acceptable (Schmitt, 1996).

Chronic physical conditions. Chronic physical conditions were assessed as the total number of chronic conditions the participant indicated having experienced in the past 12 months. The presence of thirteen total chronic conditions was evaluated, such as HIV or AIDS, neurological disorders, high blood pressure, diabetes, and obesity. Possible scores ranged from 0 to 13.

Instrumental activities of daily living. Seven items were used to evaluate how much participants’ health limits them in various activities, such as running, lifting or carrying groceries, and climbing several flights of stairs. Responses ranged from 1 = a lot to 4 = not at all. Items were reverse-coded before calculating a mean score. Higher scores indicate more difficulty in engaging in the activities.

Self-rated physical health. Self-rated physical health was assessed using a single item; participants were asked to rate their physical health from 1 = excellent to 5 = poor. Responses were coded for the analyses such that higher scores reflect better physical health.

Covariates. We included several covariates that have been shown to be linked with family functioning and health outcomes, including gender (Whisman & Sbarra, 2012), age (K. S. Birditt et al., 2005), and education (Ryan et al., 2014). Gender was coded as 0 = men or 1 = women. Age in years was included as a continuous variable, and education was also a continuous variable ranging from 1 = 6th grade or less to 12 = Graduate school, doctorate, or advanced professional degree.

### 6.3. Data Analytic Strategy

A latent profile analysis was conducted using the Full Information Maximum Likelihood (FIML) estimation method on *Mplus* 8.3 (Muthén & Muthén, 2017). The models for one to seven latent classes were compared using the Akaike Information Criteria (AIC; Akaike, 1987), the Bayesian Information Criteria (BIC; Schwarz, 1978), sample size-adjusted BIC (SABIC; Sclove, 1987), Vuong-Lo-Mendell-Rubin likelihood ratio test (VLMRT) and Adjusted Lo-Mendell-Rubin likelihood ratio test (ALMRT, Lo et al., 2001), and entropy. Lower values for AIC, BIC, and SABIC indicate a better solution. However, because the AIC, BIC, and SABIC remain influenced by sample size (Marsh et al., 2009), these indicators may suggest an addition of profiles without reaching a minimum. In such cases, ‘elbow plots’ can be used to identify the optimal number of profiles (Morin et al., 2016). This is similar to the way in which scree plots are used in exploratory factor analysis (Masyn, 2013). Significant values (i.e., *p* < 0.05) for the VLMRT and ALMRT indicate that the solution with more groups is better than the solution with one fewer group, and entropy over 0.80 is considered high (Clark & Muthén, 2009). It is important to note that entropy should not be used in isolation in determining the best solution (Lubke & Muthén, 2007).

After determining the optimal number of classes, posteriori probabilities from the latent profile analysis were used to assign each participant to a single class. A chi-square test of independence was used to evaluate whether the participants from MIDUS 1 and MIDUS-R differed in terms of the proportion of each sample that was a member of each class. The associations between class membership and the psychological and physical health variables were analyzed using a multiple regression analysis. To run this model, we first dummy coded the categorical class variable; the reference group was selected based on the class that included the greatest proportion of the total sample.

## 7. Results

### 7.1. Descriptive Statistics

Means and standard deviations of the variables in this study, as well as the bivariate correlations among the variables from the MIDUS 1 sample and MIDUS-R sample, can be found in Table 1. With no exceptions, the significant correlation coefficients found from the MIDUS 1 cohort were also significant and in the same direction as the correlation coefficients from the MIDUS-R cohort. The correlations between the dimensions of family functioning were all significant and in the expected direction. For example, family support was positively linked with spouse support and negatively associated with family strain, spouse strain, and spouse disagreement.

### 7.2. Profiles of Family Functioning

A latent profile analysis was conducted for the MIDUS 1 cohort, the MIDUS-R cohort, and the combined sample (Table 2). The overall trends of the fit indices were consistent across the MIDUS 1 and MIDUS-R cohorts and the combined sample. The latent profile analyses revealed that VLMRT and ALMRT were significant for every model (*p* < 0.001), and the AIC, BIC, and SABIC of the fit indices suggest better-fitting models with an increasing number of profiles. Because the lowest values of AIC, BIC, and SABIC corresponded to the model with the highest number of profiles, plots were used to identify the four-profile solution as the point of relative plateau (available upon request). Entropy was higher for the 3-class model than the 4-class model, but entropy was high for both models (>0.80). The profiles of the 3-class solution were distinct, but over 71% of the sample belonged to a single class. The profiles in the 4-class model were distinct, and the sample was more evenly distributed across classes. Considering all the results of the latent profile analysis, we determined that the 4-class model provided the best fit to the data. Next, we examined univariate entropy values to explore which dimensions of family functioning were most useful in identifying the latent classes. Univariate entropy values for the combined sample for each dimension of family functioning were as follows: family support = 0.22, family strain = 0.22, spouse support = 0.63, spouse strain = 0.49, and spouse disagreement = 0.35.

Means, standard deviations, and group sizes for the dimensions of family functioning by latent classes are available in Table 3. The first profile was referred to as the High Functioning profile based on the high levels of family and spousal support and low levels of family strain, spousal strain, and spousal disagreement. The second profile was referred to as High Support with Elevated Strain, and this profile is characterized by high levels of family support and spousal support, although somewhat lower than the High Functioning group, as well as elevated family and spousal strain and spousal disagreement compared to the High Functioning profile. We referred to the third profile as Moderate Support with Elevated Strain. Relative to the High Functioning and High Support with Elevated Strain profiles, the Moderate Support with Elevated Strain profile had higher spousal strain and lower family support and spousal support. Spousal disagreement was, on average, similar to the spousal disagreement of the High Support with Elevated Strain profile. The Moderate Support with Elevated Strain profile had higher family strain than the High Functioning profile and slightly lower family strain than the High Support with Elevated Strain profile. The fourth profile—Low Functioning—had the highest levels of spousal strain and disagreement and the lowest levels of family support and spousal support. Although the Low Functioning profile had higher family strain than the High Functioning profile, it had slightly lower family strain than the other two profiles.

**Figure 1.**
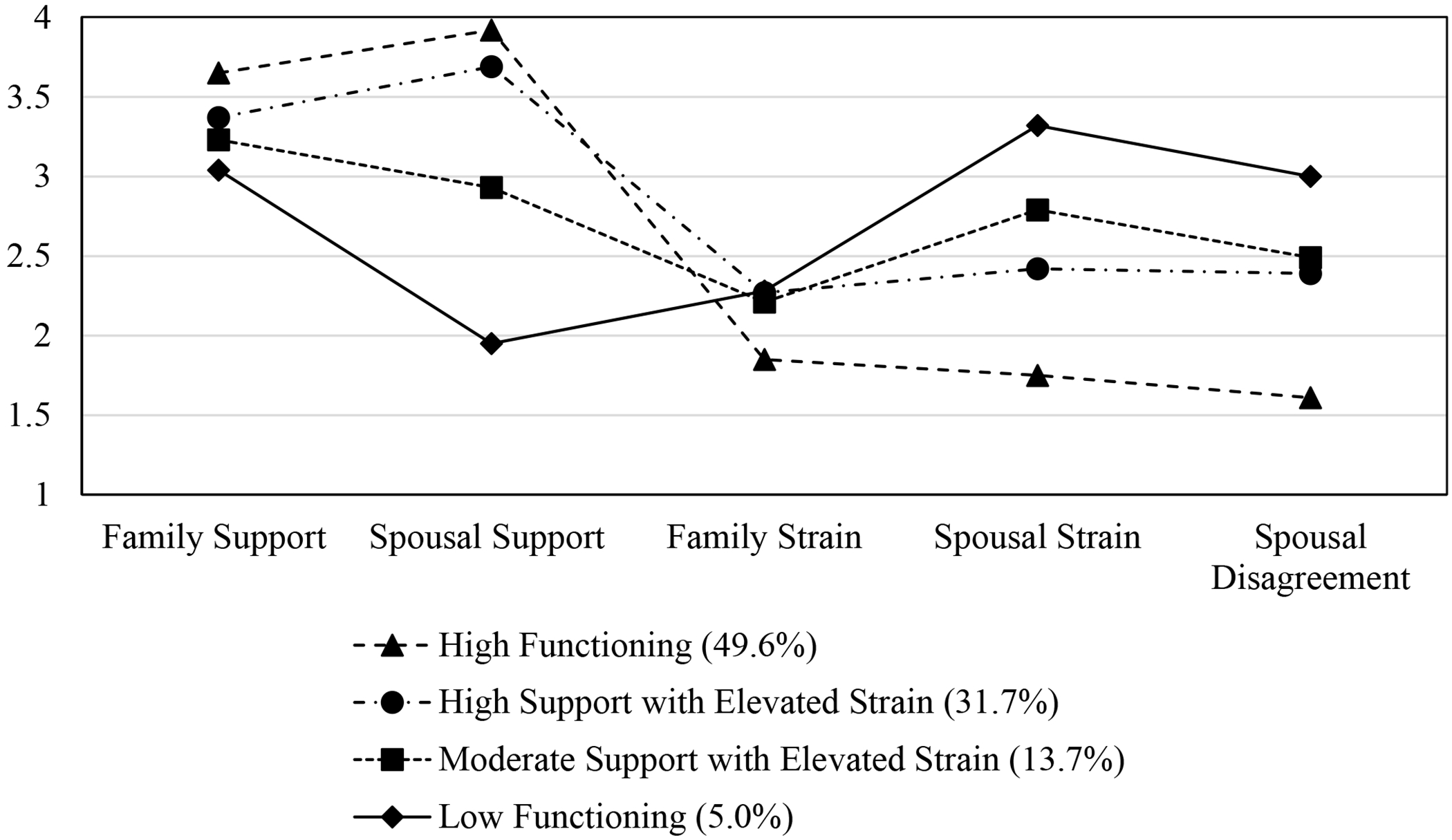
Latent classes defined by means for the combined sample.

Individuals were assigned to a single class based on their highest posterior probability. Of the 5923 participants included in the analysis, 294 (5.0%) were members of the Low Functioning class, 809 (13.7%) were members of the Moderate Support with Elevated Strain class, 2940 (49.6%) were members of the High Functioning class, and 1880 (31.7%) were members of the High Support with Elevated Strain class. The difference between the Low Functioning class, the Moderate Support with Elevated Strain class, and the High Support with Elevated Strain class was not as pronounced in the family strain dimension as it was in the other four dimensions. The mean scores for family strain had the narrowest range (0.51) of all the dimensions of family functioning (low = 1.79, high = 2.33). Spousal support had the widest range (0.98) of mean scores (low = 1.95, high = 2.93). A visual depiction of the four configurations by means of the dimensions of family functioning can be viewed in Figure 1.

### 7.3. The Relationship Between Profiles and Cohort

A chi-square test of independence showed that there was a significant association between class membership and cohort, *X*^2^ (4) = 16.29, *p* < 0.01. Further evaluation revealed that in the High Functioning class, the proportion of individuals from cohort 1 was significantly lower than the proportion of individuals from cohort 2 (*p* < 0.01). The proportion of individuals from cohort 2 in the High Support with Elevated Strain class was significantly lower than the proportion of individuals from cohort 2 (*p* < 0.05).

### 7.4. Links Between Profiles and Physical and Psychological Health Outcomes

The links between the profiles and aspects of psychological and physical health were examined using a multiple regression model. Results of this analysis, controlling for age, sex, and educational attainment, can be viewed in Table 4. Relative to membership, the High Functioning class, membership in the Moderate Support with Elevated Strain class (*β* = −0.03, *p* < 0.05), or the Low Functioning class (*β* = −0.04, *p* < 0.01) was associated with lower self-rated physical health, controlling for age, sex, and educational attainment. Compared to the High Functioning class, the Low Functioning class (*β* = −0.07, *p* < 0.001), the Moderate Support with Elevated Strain class (*β* = −0.08, *p* < 0.001), and the High Support with Elevated Strain class (*β* = −0.09, *p* < 0.001) had lower levels of instrumental activities of daily living. The High Functioning class was associated with fewer chronic conditions, relative to the Low Functioning class (*β* = 0.09, *p* < 0.001), the Moderate Support with Elevated Strain class (*β* = 0.12, *p* < 0.001), and the High Support with Elevated Strain class (*β* = 0.10, *p* < 0.001).

Compared to the other three profiles, membership in the High Functioning class was also associated with higher levels of each aspect of psychological well-being (i.e., positive relations with others, self-acceptance, autonomy, personal growth, environmental mastery, purpose in life). For example, membership in the High Functioning class was associated with increased self-acceptance, relative to membership in the Low Functioning class (*β* = −0.24, *p* < 0.001), the Moderate Support with Elevated Strain class (*β* = −0.29, *p* < 0.001), and the High Support with Elevated Strain class (*β* = −0.13, *p* < 0.001). Altogether, the model accounted for between 3% and 16% of the variance in the aspects of physical and psychological health.

## 8. Discussion

In this study, we used a pattern-based analytic approach to identify subgroups of individuals based on patterns of support and strain within their family and spousal relationships. Four profiles of family functioning emerged: High Functioning, High Support with Elevated Strain, Moderate Support with Elevated Strain, and Low Functioning. The High Functioning profile was associated with increases in markers of psychological and physical health, relative to the other three groups. Our findings, therefore, are consistent with previous research that demonstrated patterns of family functioning were related to physical and mental well-being (Oshri et al., 2015). The present study is unique, however, in that we use data from a much larger and nationally representative sample, and we also were able to include separate measures for the spousal relationship and other family relationships in the analyses.

Our use of a pattern-based approach reflects a way of thinking about family and spousal relationships that is consistent with family systems theory but different than much of the previous research involving relationships and health. Previous research that involved a variable-centered approach has pitted family relationships and intimate partnerships against each other to determine which matters more for health outcomes, but these studies have produced conflicting findings (e.g., Woods et al., 2020). Likewise, support and strain have been compared in terms of their association with health outcomes (e.g., Ryan et al., 2014). By using a pattern-based approach, we attended to the ways in which family and spousal relationships, as well as the support and strain within those relationships, exist as a part of the same systemic context, adding to the literature that highlights the relationship between families and health (e.g., Pietromonaco & Collins, 2017; Smith, 2019).

Based on the findings of this repeated cross-sectional study, policies or clinical practices that facilitate movement to the High Functioning class may bring about more positive psychological and physical health outcomes. Individuals in the High Functioning class had high support and low strain in both spousal and family relationships, so there does not appear to be a specific type of relationship or aspect of relationship that should be targeted. To move from the Low Functioning class to the High Functioning class, support needs to be increased, and strain decreased in spousal and family relationships. In accordance with the tenets of general systems theory (Bateson, 1972; von Bertalanffy, 1968), improvements in one relationship will likely galvanize changes in other relationships within the system. For example, if an adult works to improve their acrimonious relationship with their parent, then that individual may have more emotional resources to build a more supportive emotional climate in their marital relationship. With that in mind, efforts to identify and improve the most malleable relationships may contribute to movement to the High Functioning class. The findings from the present study highlight that the health of individuals should be considered in the systemic context of families. Furthermore, this study advances knowledge regarding family functioning and its association with markers of healthy aging in the manifest forms of psychological and physical well-being.

Because we utilized data from two demographically identical cohorts that were recruited nearly 20 years apart, we were able to examine the role of time period in this study. The proportion of the cohort recruited in 1995 and 1996 that belonged to the High Functioning class was less than the proportion of the cohort recruited between 2011 and 2014. This finding was unexpected because the cohort recruited between 2011 and 2014 had just experienced the Great Recession (2007 to 2009), which created a challenging economic environment for many families. Economic cycles underlying everyday life may contribute to shifts in family dynamics (Drewelies et al., 2018), and the economic conditions that the cohort recruited between 2011 and 2014 had experienced likely made it more difficult to have high-quality relationships with family members. In fact, during economic hardships, family and spousal conflict may increase, and family members may even be more likely to become violent or abusive toward each other (Schneider et al., 2016). However, it is possible that the individuals in the cohort recruited between 2011 and 2014 were more likely to have been divorced because support and strain in their marital relationship was not optimal; these divorced individuals would not have met the inclusion criteria for the present study, possibly leaving a greater proportion of individuals in the High Functioning class than there would have been if fewer individuals had gotten a divorce. Ultimately, further exploration of historical changes in patterns of family functioning and the factors that contribute to them is needed.

## 9. Limitations and Future Directions

Several limitations should be considered when interpreting the findings from the present study. For example, the alpha coefficients for the six dimensions of psychological well-being were below 0.70, which is often used as a cut-off. However, it is important to note that for measures with few items, as is the case for the dimensions of psychological well-being (three items for each dimension), lower alpha coefficients may not be problematic if the items meaningfully cover the domain of interest (Schmitt, 1996). Another limitation of the present study is that there are other dimensions of family functioning that were not assessed. Olson’s (2011) circumplex model includes flexibility, for example, which refers to the ability of the family to adjust its rules and relationship patterns in response to successfully adapt to changes, as a component of family functioning. Future research that implements a pattern-based approach to family functioning may explore whether similar profiles emerge when additional dimensions are assessed and included in the analyses. The use of self-reported data from a single timepoint should also be considered when interpreting the findings from the present study and when exploring areas for future research. Future research that links self-report data to coded behaviors within family and spousal interactions will advance understanding of family functioning. In addition, future research that utilizes longitudinal data could be used to conduct a latent transition analysis to help evaluate the stability of the profiles of family functioning across time and explore whether changes in class membership predict changes in physical and psychological health.

## 10. Conclusions

The limitations notwithstanding, this study leveraged data from two demographically identical, nationally representative samples recruited nearly 20 years apart to identify latent subgroups of family functioning and examine their links with health outcomes, thus helping to elucidate the role of family functioning in adult health. In addition, this study underscores the importance of the systemic context of families in understanding how close relationships influence physical and psychological health. It also highlights the need to consider how individuals and families change over time in response to evolving social and historical contexts.

## Figures and Tables

**Table 1.** Intercorrelations between model variables and descriptive statistics from MIDUS 1 and MIDUS-R samples.

	1	2	3	4	5	6	7	8	9	10	11	12	13	14
1. Family support	–	−0.37 *	0.28 *	−0.23 *	−0.13 *	0.15 *	0.37 *	0.07 *	0.23 *	0.23 *	0.31 *	−0.08 *	−0.01	−0.01
2. Family strain	−0.39 *	–	−0.12 *	0.29 *	0.19 *	−0.08 *	−0.23 *	−0.14 *	−0.25 *	−0.12 *	−0.22 *	0.11 *	−0.02	−0.01
3. Spousal support	0.28 *	−0.17 *	–	−0.64 *	−0.45 *	0.15 *	0.30 *	0.14 *	0.28 *	0.24 *	0.39 *	−0.09 *	0.01	0.06
4. Spouse strain	−0.21 *	0.31 *	−0.66 *	–	0.59 *	−0.12 *	−0.29 *	−0.16 *	−0.33 *	−0.20 *	−0.36 *	0.11 *	0.01	−0.05
5. Spouse disagreement	−0.17 *	0.23 *	−0.46 *	−0.58 *	–	−0.14 *	−0.26 *	−0.18 *	−0.30 *	−0.20 *	−0.30 *	0.09 *	0.00	−0.01
6. Purpose in life	0.18 *	−0.11 *	0.16 *	−0.14 *	−0.11 *	–	0.31 *	0.24 *	0.33 *	0.52 *	0.33 *	−0.16 *	0.01	−0.02
7. Relationships with others	0.36 *	−0.20 *	0.27 *	−0.26 *	−0.22 *	0.35 *	2212	0.22 *	0.44 *	0.50 *	0.53 *	−0.10 *	0.00	−0.02
8. Autonomy	0.11 *	−0.13 *	0.12 *	−0.13 *	−0.13 *	0.14 *	0.23 *	–	0.38 *	0.34 *	0.29 *	−0.08 *	−0.01	0.02
9. Environmental mastery	0.25 *	−0.26 *	0.28 *	−0.31 *	−0.26 *	0.24 *	0.36 *	0.32 *	–	0.50 *	0.62 *	−0.25 *	0.00	0.01
10. Personal growth	0.20 *	−0.09 *	0.19 *	−0.13 *	−0.10 *	0.39 *	0.40 *	0.26 *	0.36 *	–	0.57 *	−0.18 *	−0.01	−0.01
11. Self-acceptance	0.28 *	−0.23 *	0.31 *	−0.30 *	−0.23 *	0.34 *	0.48 *	0.30	0.57 *	0.42 *	–	−0.23 *	0.01	0.01
12. Chronic conditions	−0.08 *	0.13 *	−0.13 *	0.16 *	0.08 *	−0.17 *	−0.18 *	−0.08 *	−0.24 *	−0.16 *	−0.25 *	–	−0.02	−0.01
13. Self-rated physical health	−0.02	0.01	0.01	−0.01	0.00	0.01	0.03	0.01	0.00	−0.01	0.00	0.02	–	0.52 *
14. Activities of daily living	−0.02	0.01	−0.01	0.00	−0.02	0.01	0.01	0.00	−0.03	−0.01	−0.01	−0.02	0.53 *	–
*M* (cohort 1)	3.47	2.07	3.60	2.22	2.06	5.62	5.56	5.47	5.43	5.98	5.66	2.26	2.48	1.64
*SD* (cohort 1)	0.58	0.59	0.54	0.61	0.73	1.16	1.30	1.16	1.12	1.01	1.10	2.37	1.02	0.83
*M* (cohort 2)	3.47	1.99	3.63	2.11	2.00	5.62	5.58	5.42	5.58	5.57	5.53	2.50	2.47	1.60
*SD* (cohort 2)	0.60	0.63	0.53	0.63	0.71	1.05	1.23	1.01	1.08	1.06	1.20	2.53	1.00	0.79

*Note.* MIDUS 1 correlation coefficients are below the diagonal and MIDUS-R correlation coefficients are above the diagonal. *M* = mean, *SD* = standard deviation.

* *p* < 0.001 (two-tailed).

**Table 2.** Model fit statistics for the one- through seven-class latent profile analysis solutions.

Sample	*k*	AIC	BIC	SABIC	Entropy	VLMRT *p*	ALMRT *p*
Combined Sample							
	1	54,620.0	54,686.8	54,655.1	-	-	-
	2	48,527.1	48,634.1	48,583.1	0.915	<0.001	<0.001
	3	46,042.8	46,189.9	46,120.0	0.897	<0.001	<0.001
	4	44,471.1	44,658.2	44,569.2	0.806	<0.001	<0.001
	5	43,555.6	43,783.0	43,674.1	0.817	<0.001	<0.001
	6	42,824.6	43,092.1	42,965.0	0.824	<0.001	<0.001
	7	42,284.9	42,592.5	42,446.3	0.812	<0.001	<0.001
Cohort 1							
	1	38,931.2	38,994.7	38,962.6	-	-	-
	2	34,584.5	34,686.2	34,635.3	0.910	<0.001	<0.001
	3	32,819.9	32,959.7	32,889.8	0.897	<0.001	<0.001
	4	31,675.9	31,853.8	31,764.8	0.808	<0.001	<0.001
	5	31,099.6	31,304.6	31,196.5	0.814	<0.001	<0.001
	6	30,515.5	30,769.6	30,642.5	0.821	<0.001	<0.001
	7	30,188.4	30,480.7	30,334.5	0.828	<0.001	<0.001
Cohort 2							
	1	15,625.3	15,679.6	15,647.8	-	-	-
	2	13,873.4	13,960.3	13,909.4	0.929	<0.001	<0.001
	3	13,158.7	13,278.0	13,208.1	0.910	<0.001	<0.001
	4	12,750.5	12,902.9	12,813.4	0.800	<0.001	<0.001
	5	12,421.9	12,606.4	12,498.3	0.827	<0.001	<0.001
	6	12,213.6	12,430.7	12,303.6	0.812	<0.001	<0.001
	7	12,028.5	12,278.1	12,132.0	0.812	<0.001	<0.001

*Note*. *k* = number of classes; AIC = Akaike Information Criteria; BIC = Bayesian Information; SABIC = sample size-adjusted BIC; VLMRT = Vuong-Lo-Mendell-Rubin Likelihood Ratio Test; ALMRT = Adjusted Lo-Mendell-Rubin Likelihood Ratio Test.

**Table 3.** Means, standard deviations, and group sizes for the relationship variables by latent classes.

		Family Support		Spouse Support		Family Strain		Spouse Strain		Disagreement		

Class	Cohort	*M*	*SD*	*M*	*SD*	*M*	*SD*	*M*	*SD*	*M*	*SD*	*n*(%)
1	1	3.66	0.20	3.93	0.02	1.85	0.27	1.77	0.12	1.59	0.23	2037 (48.0)
1	2	3.63	0.22	3.92	0.02	1.79	0.30	1.68	0.14	1.59	0.23	903 (53.8)
2	1	3.36	0.35	3.68	0.05	2.31	0.32	2.46	0.12	2.40	0.35	1388 (32.7)
2	2	3.34	0.40	3.68	0.05	2.25	0.34	2.47	0.14	2.39	0.35	492 (29.3)
3	1	3.24	0.40	2.92	0.06	2.21	0.33	2.79	0.21	2.50	0.45	603 (14.2)
3	2	3.21	0.43	2.89	0.06	2.17	0.48	2.76	0.20	2.50	0.46	206 (12.3)
4	1	3.08	0.65	1.95	0.13	2.33	0.46	3.33	0.24	3.03	0.55	216 (5.1)
4	2	3.00	0.57	1.95	0.13	2.15	0.58	3.27	0.22	2.90	0.56	78 (4.6)

*Note*. *M* = mean, *SD* = standard deviation. Class 1 = High Functioning, Class 2 = High Support with Elevated Strain, Class 3 = Moderate Support with Elevated Strain, Class 4 = Low Functioning.

**Table 4.** Standardized regression coefficients for the model with class membership predicting indicators of physical and mental health.

Predictor	Purpose in Life	Relation with Others	Autonomy	Environ. Mastery	Personal Growth	Self Acceptance	Chronic Conditions	Self-Rated Physical Health	Activities of Daily Living
Low Functioning	−0.12 ***	−0.22 ***	−0.07 ***	−0.20 ***	−0.06 ***	−0.24 ***	0.09 ***	−0.03 *	−0.07 ***
Mod. Support, Elev. Strain	−0.15 ***	−0.27 ***	−0.13 ***	−0.25 ***	−0.10 ***	−0.29 ***	0.12 ***	−0.04 **	−0.08 ***
High Support, Elev. Strain	−0.13 ***	−0.21 ***	−0.11 ***	−0.22 ***	−0.07 ***	−0.20 ***	0.10 ***	−0.02	−0.09 ***
Age	−0.14 ***	0.01	0.07 ***	0.08 ***	0.12 ***	−0.02	0.21 ***	−0.15 ***	−0.32 ***
Education	0.21 ***	0.09 ***	0.02	0.12 ***	0.29 ***	0.16 ***	−0.12 ***	−0.04 **	0.16 ***
Sex	0.01	0.16 ***	−0.04 ***	−0.02	0.03 **	0.02	0.12 ***	0.00	−0.11 ***

*Note.* The High Functioning class is the reference group.

* *p* < 0.01.

** *p* < 0.01

*** *p* < 0.001 (two-tailed).

## Data Availability

Data available in a publicly accessible repository at https://midus.colectica.org/Account/Login?returnUrl=%2F (accessed on 23 April 2026).
